# Extracranial doses in stereotactic and conventional radiotherapy for pituitary adenomas

**DOI:** 10.1120/jacmp.v7i2.2203

**Published:** 2006-05-25

**Authors:** Thomas Samuel Ram, Paul B. Ravindran, Faith R. Viswanathan, Perungulam Narayan Viswanathan, Simon P. Pavamani

**Affiliations:** ^1^ Christian Medical College Department of Radiation Oncology Vellore 632 004 India

**Keywords:** stereotactic radiotherapy, extracranial dose, thermoluminscent dosimetry, pituitary adenoma

## Abstract

The purpose of this study is to determine the extracranial dose in patients treated for pituitary adenoma with conventional and stereotactic radiotherapy (SRT). Twelve patients receiving treatment with radiation for pituitary adenoma were selected. Six patients underwent SRT, and six patients underwent conventional radiotherapy. Extracranial doses were measured with pre‐irradiation annealed lithium fluoride thermoluminscent dosimetry (TLD) chips. The chips were wrapped and placed on the patients’ skin, over each eyelid, the thyroid, chest, and scrotum for males and over the suprapubic region for females. Postradiation annealing was done, and the TLDs were read in a TLD reader system. The results were analyzed using the Wilcoxon matched‐pairs signed rank test by SPSS, version 6.01. The doses to the thyroid, center, and gonads were significantly higher (74.62±9.12mrad, 65.42±9.35mrad, and 58.42±5.36mrad, respectively) in patients receiving SRT than in conventional radiotherapy portals (69.45±21.19mrad, 38.33±19.44mrad, and 31.41±18.25mrad). But the average doses to the right eye (84.84±8.80mrad) and to the left eye (85.68±5.82mrad) in the stereotactic group were less when compared with the patients treated with conventional radiotherapy, who received 127.5±37.90mrad and 117.29±34.01mrad, respectively. In conclusion, SRT is definitely superior to conventional radiotherapy as far as dose to the surrounding normal structures is concerned. The higher extracranial doses in SRT seem to be within the acceptable range; however, the clinical significance of this is still unclear and needs longer followup.

PACS number: 87.53.Ly

## I. INTRODUCTION

Pituitary tumors represent 12% of all intracranial tumors.^(1)^ Most of these tumors are histologically benign but are capable of causing significant morbidity to the patient because of their strategic anatomical location and function.

Fixed‐beam radiotherapy using cobalt‐60 γ‐rays or photons from a linear accelerator has been the time‐tested modality of treatment for pituitary adenomas. But this modality is associated with increased morbidity of acute effects and late effects such as temporal lobe necrosis. This is due to the significant amount of dose delivered to a given part of the brain as a result of the fixed beams and the nature of the surrounding normal tissue. With the availability of precision radiation therapy techniques, it has been possible to slightly improve the overall disease‐free survival and decrease the morbidity.

In 1951, Leksell^(2)^ and Larsson developed the concept of radiosurgery. Later, in 1968, a special apparatus, called the Gamma Knife, was developed by Leksell.^(3)^ Today, Leksell's technique is used as an effective treatment for many conditions such as arteriovenous malformations, pituitary tumors, acoustic neuromas, craniopharyngiomas, meningiomas, metastatic and skull base tumors, and primary brain tumors.

Stereotactic radiotherapy (SRT) has been used extensively in the management of the pituitary tumor because of the tumor's small dimensions and the ability to consistently reproduce the treatment setup. Various trials have compared the efficacy and morbidity of fractionated SRT with stereotactic radiosurgery.^(^ ^4^ ^,^ ^5^ ^)^ Stereotactic radiosurgery (SRS) is no longer the preferred treatment because of the morbidity associated with it and its deleterious effects on the longtime survival of patients with pituitary adenoma; SRT is now the standard recommended modality^(6)^ in eligible patients. Where SRT is not possible, either because the size of the tumor is more than 4 to 5 cm, the technology is not available, or because of restricted finances, cobalt‐60 γ‐rays or 6‐MV or 15‐MV photon beams are used to deliver conventional three‐field parallel, opposed, or arc therapy. In a developing country such as India, where there are only a few linear accelerators and a few centers equipped with the technology to perform SRT, there was a need to compare the two different modalities to develop guidelines for referral for SRT. In our institute, where both modalities are available, this would help in deciding the optimal treatment for each patient. To date, there is not enough literature comparing these two modalities.

This study was undertaken to compare the extracranial doses of SRT and conventional RT in the management of pituitary adenoma.

## II. MATERIALS AND METHODS

Twelve patients diagnosed with pituitary adenoma were selected. Six patients underwent SRT, and six patients underwent conventional radiotherapy. Extracranial doses were measured in all patients. Cone‐based SRT was delivered using a 6‐MV linear accelerator. Radionics X‐Knife version 3 planning software was used for treatment planning. A dose of 50.4 Gy to 90% isodose in 28 fractions was prescribed for patients undergoing SRT, and a similar dose was prescribed to those patients undergoing conventional radiotherapy. Lithium fluoride thermoluminescent dosimeter (TLD) chips with dimensions 3mm×3mm×3mm (7.5% Li‐6 and 92.5% Li‐7; Harshaw TLD‐100) were used. The TLDs were calibrated with known low‐exposure doses with 1.5 mm Perspex buildup, and the calibration curve was thus obtained between the known dose and the TLD output. These calibrated TLDs were used for patient measurement. Pre‐irradiation annealing was done by heating the chips at 400 °C for 1 h. The chips were wrapped and placed on the patient's skin over both eyelids, thyroid, chest, and gonads. Postradiation annealing was done at 100 °C for 10 min. After postradiation annealing, the TLDs were read in a TLD reader system (Semi‐automatic Teledyne Brown Engineering, USA). Statistical analysis was done using the Wilcoxon matched‐pairs signed rank test by SPSS, version 6.01.

## III. RESULTS

The extracranial sites such as the eyes and the thyroid, which were anatomically more proximal to the volume irradiated, did not show a significant difference in the doses measured. The average dose to the right eye was 84.84±8.80mrads for SRT and 127.5±37.90mrad for conventional RT. This was not statistically significant (p=0.05). The average dose to the left eye was 85.68±5.82mrad for SRT and 117.29±34.01mrad for conventional RT. This also was not statistically significant (p>0.05). The average dose to the thyroid was 74.62±9.12mrad for SRT and 69.45±21.19mrad for conventional RT. This, too, was not statistically significant (p>0.05). (See Tables 1 and 2.)

**Table 1:** TLD readings: Stereotactic radiation therapy

Patient	Right eye	Dose (mrad)	Left eye	Dose (mrad)	Thyroid	Dose (mrad)	Center	Dose (mrad)	Gonads	Dose (mrad)
patient 1	2.68	75.04	2.78	77.84	2.61	73.08	2.6	72.8	2.26	63.28
patient 2	3.04	85.12	3.09	86.52	2.04	57.12	2.73	76.44	2.0	56.00
patient 3	2.98	83.44	3.37	94.36	2.89	80.92	2.04	57.12	1.94	54.32
patient 4	2.95	82.6	3.06	85.68	2.74	76.72	2.43	68.04	2.25	63.00
patient 5	3.62	101.36	3.17	88.76	2.92	81.76	1.85	51.8	1.82	50.96
patient 6	2.91	81.48	2.89	80.92	2.79	78.12	2.37	66.36	2.25	63.00
mean	3.03	**84.84**	3.06	**85.68**	2.67	**74.62**	2.34	**65.43**	2.09	**58.43**
SD	0.31	**8.80**	0.21	**5.83**	0.33	**9.12**	0.33	**9.36**	0.19	**5.36**

**Table 2:** TLD Readings: Conventional radiation therapy

Patient	Right eye	Dose (mrad)	Left eye	Dose (mrad)	Thyroid	Dose (mrad)	Center	Dose (mrad)	Gonads	Dose (mrad)
patient 1	4.99	124.75	5.09	127.25	4.12	103	2.48	62	1.96	49
patient 2	5.1	127.5	4.3	107.5	2.4	60	0.95	23.75	0.67	16.75
patient 3	5.65	141.25	4.93	123.25	1.93	48.25	0.64	16	0.42	10.5
patient 4	7.25	181.25	6.68	167	2.22	55.5	0.96	24	0.7	17.5
patient 5	2.54	63.5	2.47	61.75	2.49	62.25	2.25	56.25	1.92	48
patient 6	5.07	126.75	4.68	117	3.51	87.75	1.92	48	1.87	46.75
mean	5.10	**127.50**	4.69	**117.29**	2.78	**69.46**	1.53	**38.33**	1.26	**31.42**
SD	1.52	**37.90**	1.36	**34.02**	0.85	**21.19**	0.78	**19.45**	0.73	**18.25**

As measurement site of the extracranial dose moved farther away from the volume irradiated, the difference became quite apparent. This is due to the inclination of arcs in SRT more toward the sagittal plane. The average dose to the center was 65.42±9.35mrad for SRT and 38.33±19.44mrad for conventional RT, which was statistically significant (p<0.05). The average dose to the gonads was 58.42±5.36mrad for SRT and 31.41±18.25mrad for conventional RT, which also was statistically significant (p<0.05). (See Table 3.) The dose (in millirads) was calculated by multiplying the reading obtained for each organ of interest and the total number of fractions delivered in each respective modality.

**Table 3:** Extracranial doses (TLD measurements summary)

	SRT (mrad) (SD)	Conventional (mrad) (SD)	*p*‐ Value
right eye	84.84 (8.80)	127.5 (37.90)	0.05
left eye	85.68 (5.83)	117.29 (34.02)	0.07
thyroid	74.62 (9.12)	69.46 (21.19)	0.60
center	65.43 (9.36)	38.33 (19.45)	0.04
gonads	58.43 (5.36)	31.42 (18.25)	0.03

## IV. DISCUSSION

**Figure 1:**
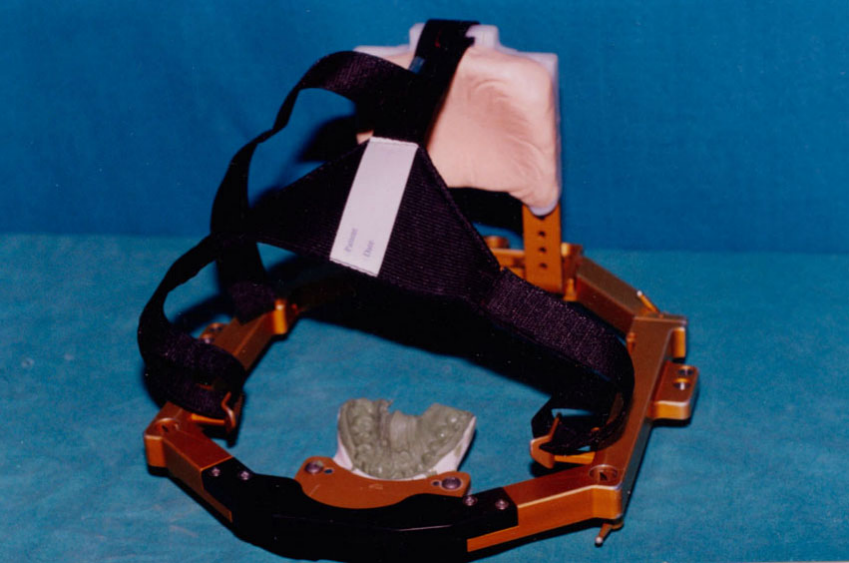
Gill‐Thomas‐Cosman frame used for stereotactic radiotherapy

In SRT, the presence of the frame (Fig. 1) makes it necessary to choose beams passing in the craniocaudal direction.

Because of the inclination of the arcs in SRT toward the midsagittal plane, the doses to midline structures such as the gonads were significantly higher than in conventional RT. Kim et al.^(7)^ reported that extracranial doses from SRT were less than 1.5% of the maximum doses in the intracranial target. Although the benefit with SRT is considerably greater than the estimated risk in the extracranial regions, it would be prudent to keep the dose to extracranial normal tissue as low as possible. This can be achieved by avoiding any direct beam passing through the critical organs such as the thyroid and the breast. If this is not possible, modifying the arc angle, adding a smaller arc, or reducing the weightage for the arc can reduce the exit dose.

## V. CONCLUSION

Stereotactic radiotherapy is definitely superior to conventional RT as far as dose to the surrounding normal structures is concerned. Owing to the spatial orientation of the arcs, the extracranial doses in SRT are marginally greater than in conventional radiotherapy. Although the doses seem to be within the acceptable range, their clinical significance is still unclear and needs more followup.
